# Mitochondrial ASncmtRNA-1 and ASncmtRNA-2 as potent targets to inhibit tumor growth and metastasis in the RenCa murine renal adenocarcinoma model

**DOI:** 10.18632/oncotarget.18460

**Published:** 2017-06-13

**Authors:** Vincenzo Borgna, Jaime Villegas, Verónica A. Burzio, Sebastián Belmar, Mariela Araya, Emanuel Jeldes, Lorena Lobos-González, Verónica Silva, Claudio Villota, Luciana Oliveira-Cruz, Constanza Lopez, Teresa Socias, Octavio Castillo, Luis O. Burzio

**Affiliations:** ^1^ Andes Biotechnologies SpA, Santiago, Chile; ^2^ Fundación Ciencia & Vida, Ñuñoa, Santiago, Chile; ^3^ Servicio de Urología, Hospital Barros Luco-Trudeau, Universidad de Santiago, Santiago, Chile; ^4^ Facultad de Ciencias Biológicas, Universidad Andrés Bello, Santiago, Chile; ^5^ Facultad de Salud, Universidad Bernardo O Higgins, Santiago, Chile; ^6^ Centro de Cirugía Robótica, Clínica Indisa, Santa María, Santiago, Chile

**Keywords:** murine renal cancer, ncRNA, mitochondria, antisense therapy

## Abstract

Knockdown of antisense noncoding mitochondrial RNAs (ASncmtRNAs) induces apoptosis in several human and mouse tumor cell lines, but not normal cells, suggesting this approach for a selective therapy against different types of cancer. Here we show that *in vitro* knockdown of murine ASncmtRNAs induces apoptotic death of mouse renal adenocarcinoma RenCa cells, but not normal murine kidney epithelial cells. In a syngeneic subcutaneous RenCa model, treatment delayed and even reversed tumor growth. Since the subcutaneous model does not reflect the natural microenviroment of renal cancer, we used an orthotopic model of RenCa cells inoculated under the renal capsule. These studies showed inhibition of tumor growth and metastasis. Direct metastasis assessment by tail vein injection of RenCa cells also showed a drastic reduction in lung metastatic nodules. *In vivo* treatment reduces survivin, N-cadherin and P-cadherin levels, providing a molecular basis for metastasis inhibition. In consequence, the treatment significantly enhanced mouse survival in these models. Our results suggest that the ASncmtRNAs could be potent and selective targets for therapy against human renal cell carcinoma.

## INTRODUCTION

Renal cell carcinoma (RCC) constitutes almost 3% of all malignant tumors in adults. According to recent statistics, 62,700 new cases and 14,240 deaths by RCC in female and male patients were expected in the United States in 2016 [1]. The overall 5-yr survival rate of localized RCC patients is about 93% and standard therapy is partial or radical nephrectomy [1]. Similar statistics have been reported in Europe [2, 3]. Metastatic RCC (mRCC) holds a poor prognosis and patients face limited therapeutic options [4]. The first-line treatment for mRCC is the multi-tyrosine kinase inhibitor sunitinib, which displays only a modest therapeutic potential [5]. Several other treatments have been described, such as the mTOR inhibitor temsirolimus [6] and high-dose IL-2 [7], which also display low efficacy, in addition to high toxicity. Immunotherapy with a monoclonal antibody against VEFG1 and VEFG2 (VEGF-Trap) showed a significant reduction of 74% in tumor size, but without complete tumor remission [8]. On the other hand, combined therapy with histone deacetylase inhibitors and high-dose IL-2 reduced tumor growth from 1900 mg to 200 mg considering however the toxicity of IL-2 [9]. Sequential treatment with sunitinib followed by everolimus in an orthotopic RenCa model led to a modest reduction in both primary tumors and metastatic foci. These effects were not observed with sunitinib alone or followed by sorafenib, which are currently used as first- and second-line therapies against mRCC [10]. Therefore, there is an urgent need to find new non-toxic and effective therapeutic approaches for mRCC.

Human cells express a unique family of sense (SncmtRNA) and antisense ASncmtRNAs) non-coding mitochondrial RNAs [11–13]. The SncmtRNA is expressed in normal proliferating cells and tumor cells, but not in resting cells, suggesting a role for this transcript in cell proliferation [11, 12]. Normal proliferating cells also express two antisense transcripts: ASncmtRNA-1 and ASncmtRNA-2 [12]. Remarkably however, the ASncmtRNAs are downregulated in human and mouse tumor cell lines, including mouse RenCa cells [12, 14]. Thus, it seems that at least in these two mammalian species, downregulation of the ASncmtRNAs seems to be an important step in carcinogenesis and represents a new and generalized pro-tumorigenic hallmark of cancer [15].

Knockdown of human ASncmtRNAs (ASK for short) *in vitro* using antisense oligonucleotides (ASOs) induces apoptotic cell death of a wide array of human cancer cell lines from several tissue origins [16]. Similarly, we reported recently that ASK *in vitro* also induces apoptotic cell death of the aggressive murine melanoma cell line B16F10, together with downregulation of survivin, an important member of of the AIP family [14, 16–21]. Moreover, using a syngeneic subcutaneous B16F10 melanoma model, we reported that ASK induces a drastic inhibition of tumor growth and lung and liver metastasis suggesting that the ASncmtRNAs are potent targets to develop a new treatment for melanoma [14]. However, oligonucleotides are not able to enter mitochondria *in vivo* [22, 23]. Therefore, the effective effect of ASO in cells and *in vivo* is because, in human and murine tumor and normal cells, the SncmtRNA and the ASncmtRNAs exit the mitochondria and are found localized in the cytoplasm and the nucleus [24].

Here we show that ASK induces apoptotic cell death in the RenCa murine RCC cell line. Translation of these results to *in vivo* syngeneic RCC assays (subcutaneous, orthotopic and tail vein inoculation), showed that ASK inhibits tumor growth and lung metastasis, suggesting that the ASncmtRNAs might be potent targets for human RCC therapy.

## RESULTS

### Expression of the mitochondrial lncRNAs

**Figure 1:**
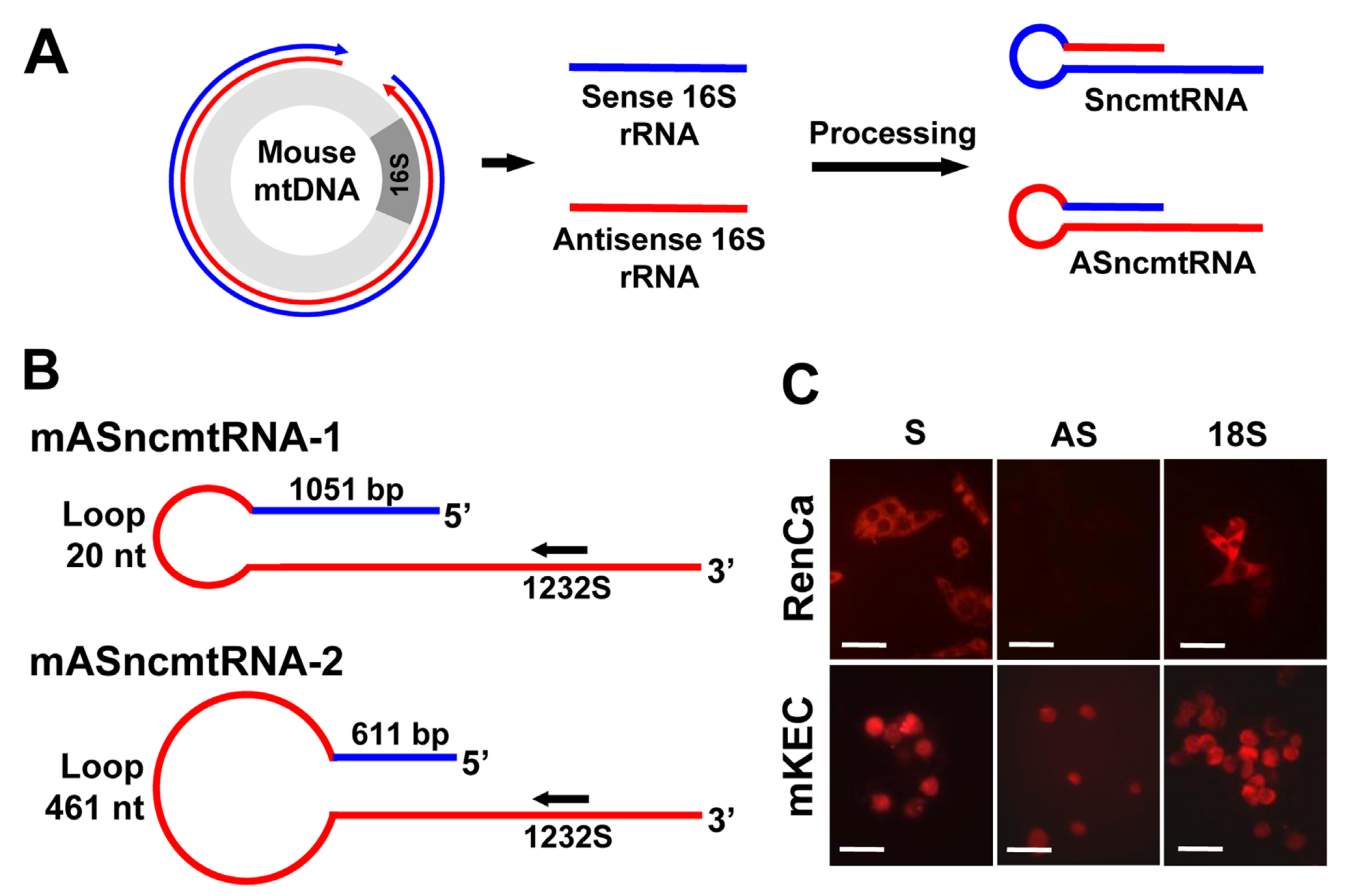
Expression of the mSncmtRNA and the mASncmtRNAs in normal mouse kidney epithelial cells (mKEC) and RenCa cells **A**. Scheme depicting the putative origin of the mouse ncmtRNAs. Segments generated from bidirectional transcription of the 16S region of the mouse mtDNA are processed to give rise to the SncmtRNA and the ASncmtRNAs. In blue, heavy-strand transcript; in red, light-strand transcript. **B**. Schematic representation of the mASncmtRNA-1 and -2, indicating the size of the loop, the length of the IR and position of ASO-1232S used in this study. **C.** FISH of mSncmtRNA and the mASncmtRNAs in RenCa and mKEC cells (Bars = 25 μm).

As the human transcripts, murine ncmtRNAs should arise from the bidirectional transcription [25] of the mitochondrial genome and processing of segments from the 16S rRNA gene [11, 12]. Figure 1A shows a schematic representation of transcription of the mouse mitochondrial DNA (mtDNA) from the heavy strand promoter (blue) and the light strand promoter (red). Segments originated from the 16S gene are processed to give rise to SncmtRNA and the ASncmtRNAs (Figure 1A and 1B). A schematic of the structures of murine ASncmtRNA-1 and -2 are shown in Figure 1B [11], where the relative position of ASO-1232S, modified with phosphorothioate internucleosidic linkages [26] used in this study is indicated. Fluorescence *in situ* hybridization (FISH) showed that normal epithelial cells freshly isolated from mouse kidney (mKEC) express the SncmtRNA and the ASncmtRNAs transcripts (Figure 1C). In contrast, RenCa cells express the SncmtRNA and downregulate the ASncmtRNAs, similar to human and other mouse tumor cells (Figure 1C) [12, 14, 16].

### ASK induces inhibition of cell proliferation

**Figure 2:**
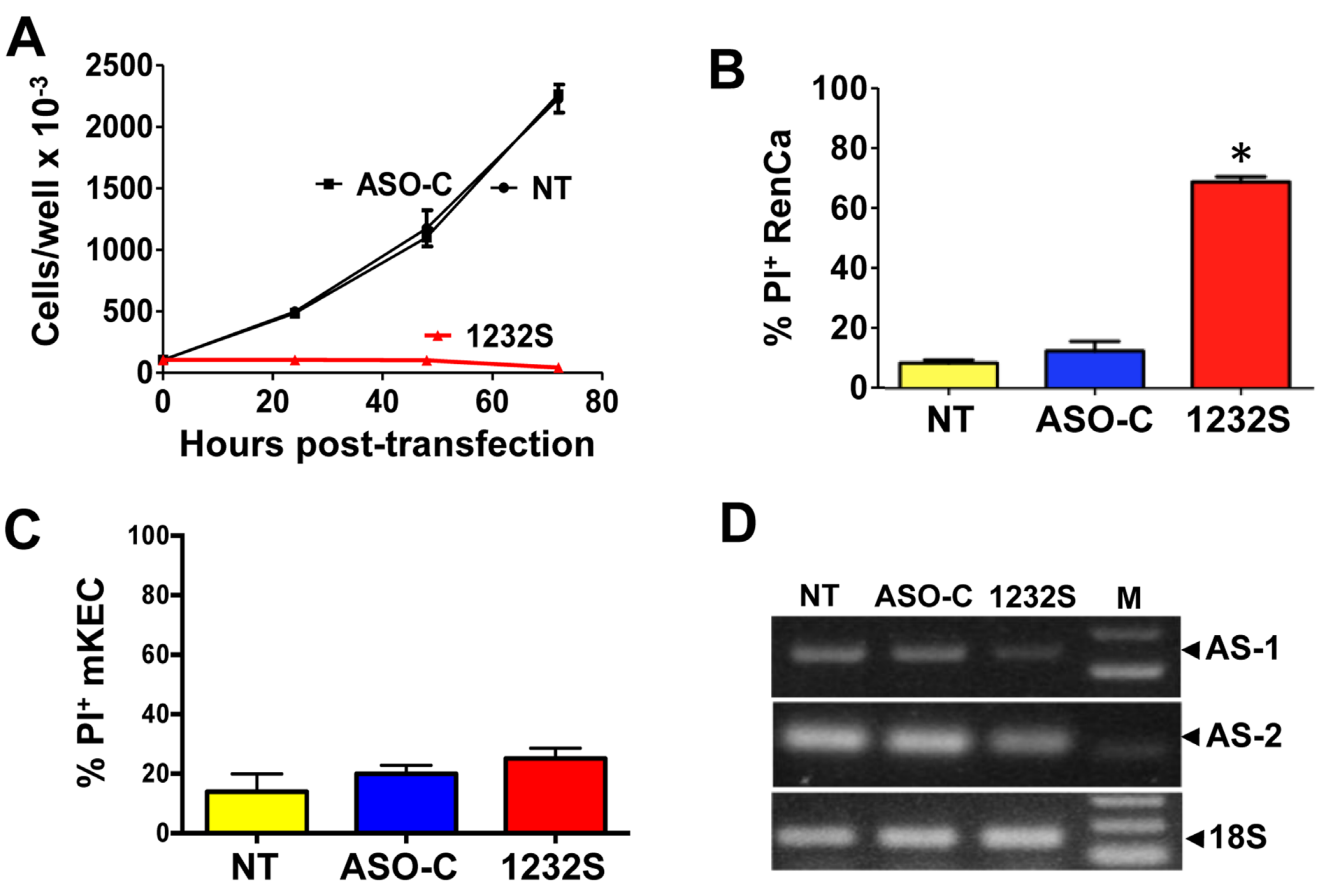
ASK induces inhibition of proliferation and death of RenCa cells **A**. RenCa cells (100,000/ well) were transfected in triplicate with 100 nM of ASO-C, or ASO-1232S or left untreated (NT). At 24, 48 and 72 h post-transfection, total cell number was determined. At 72 h, ASO-1232S induced drastic inhibition of cell proliferation compared to controls (*p < 0.005). **B**. Cells were treated as in (A) for 48 h. ASK induced over 70% cell death evaluated by PI staining and cytometric analysis (*p < 0,05). **C**. ASK of normal mKEC for 48 h does not induce significant death, compared to controls. **D**. After a 48 h treatment, knockdown of the ASncmtRNAs was confirmed by RT-PCR amplification of mASncmtRNA-1 (648 bp amplicon) and mASncmtRNA-2 (209 bp amplicon), using 18S rRNA (180 bp amplicon) as control (M, 100-bp ladder).

ASK induces a drastic inhibition of RenCa cell proliferation (Figure 2A). At 48 h post-treatment, ASO-1232S induces massive (70%) cell death, as determined by propidium iodide (PI) exclusion, compared to controls (Figure 2B). In contrast, viability of normal mKEC cells remains unaffected by the same treatment (Figure 2C). Figure 2D confirms knockdown of the ASncmtRNA-1 and -2 in RenCa cells.

### ASK induces apoptotic cell death of RenCa cells

**Figure 3:**
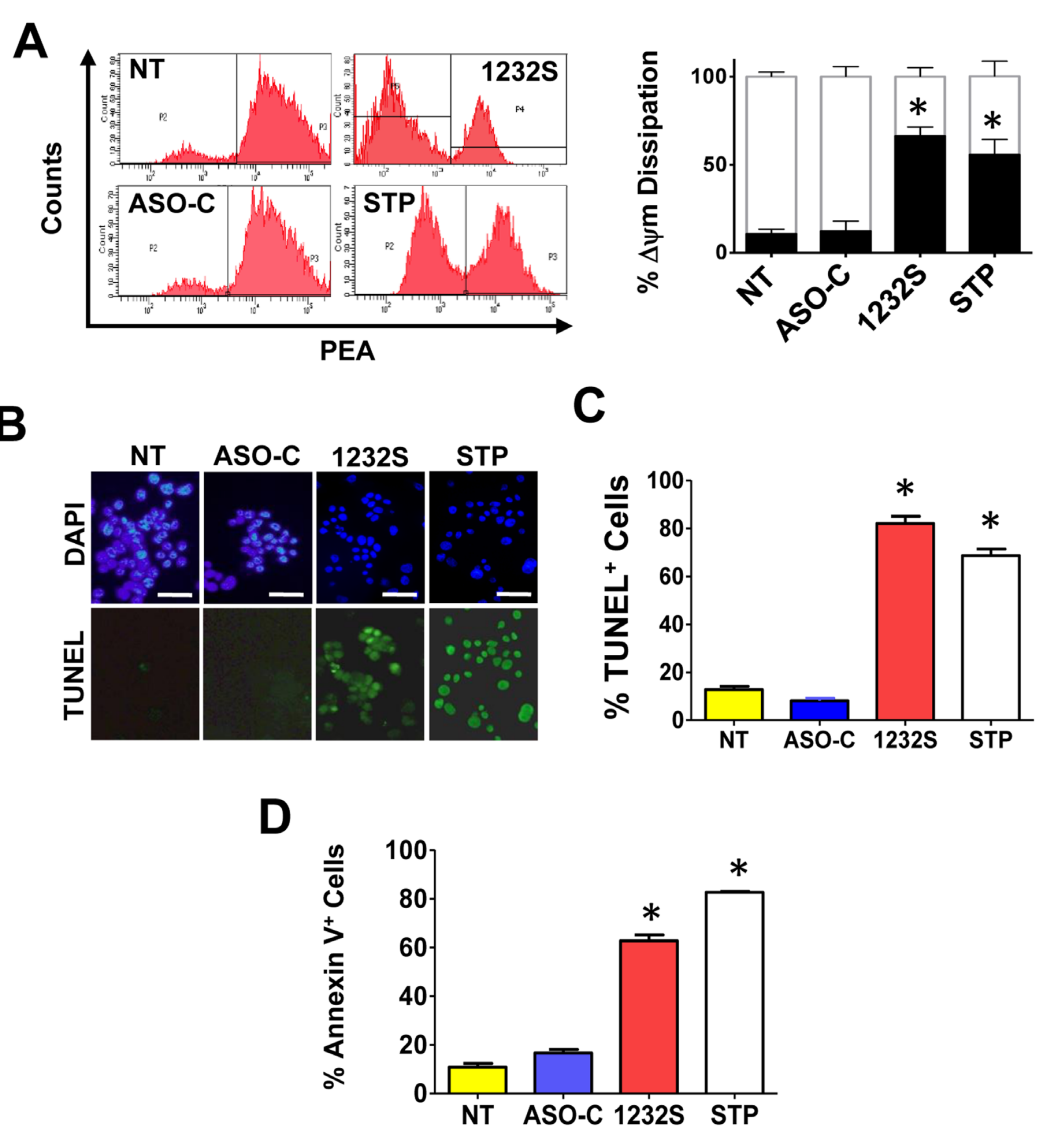
ASK induces apoptosis in RenCa cells **A**. ASK induces dissipation of Δψm. RenCa cells were transfected with 100 nM ASO-C or ASO-1232S or left untreated (NT). As positive control, a parallel culture was incubated with STP. At 24 h post-transfection, cells were harvested, stained with 20 nM TMRM for 15 min and analyzed by flow cytometry. Both ASK and STP induce dissipation of Δψm compared to controls. A triplicate analysis showed that ASK induced around 70% of depolarized cells and STP about 55%, compared to controls (*p < 0.001). **B**., **C**. ASK induces DNA fragmentation. RenCa cells treated as in (A) for at 48 h were fixed, subjected to fluorescent TUNEL assay and counterstained with DAPI (see Materials and Methods). As positive control, cells were treated with 1 μM STP. ASK and STP but not controls induced marked DNA fragmentation (Bars = 25 μm). A triplicate analysis shows that ASK and STP induce about 80% and 70% DNA fragmentation, respectively (*p < 0.05). **D**. ASK induces translocation of phosphatidylserine to the outer layer of the plasma membrane. RenCa cells treated as in (A) for 24 h were fixed and stained with Annexin-V-Alexa fluor 488 and analyzed by flow cytometry. ASK induced a drastic increase in Annexin V-positive cells, compared to controls (*p < 0.001).

Cell death by apoptosis was corroborated by different determinations [27]. One of the early stages of cell death by apoptosis is dissipation of mitochondrial membrane potential (ΔΨm) [28–30]. RenCa cells were transfected with ASO-1232S or ASO-C or left untreated or treated with staurosporine (STP) for 24 h as positive control. Cells were harvested and analyzed by flow cytometry (Materials and Methods). Treatment with STP or ASO-1232S induced a marked dissipation of ΔΨm, compared to controls (Figure 3A). Three independent experiments showed that ASK and STP induce between 50 and 60% dissipation of ΔΨm compared to around 10% in controls (Figure 3A). To determine if ASK also induces DNA fragmentation, RenCa cells were transfected for 48 h with ASO-C, or ASO-1232S or left untreated. Fluorescent TUNEL assay, using STP as positive control, shows that ASK or STP treatment induces DNA fragmentation (Figure 3B, 3C). ASK also induces translocation of phosphatidylserine to the outer layer of the plasma membrane, evidence by an increase in the Annexin V-positive population (Figure 3D).

**Figure 4:**
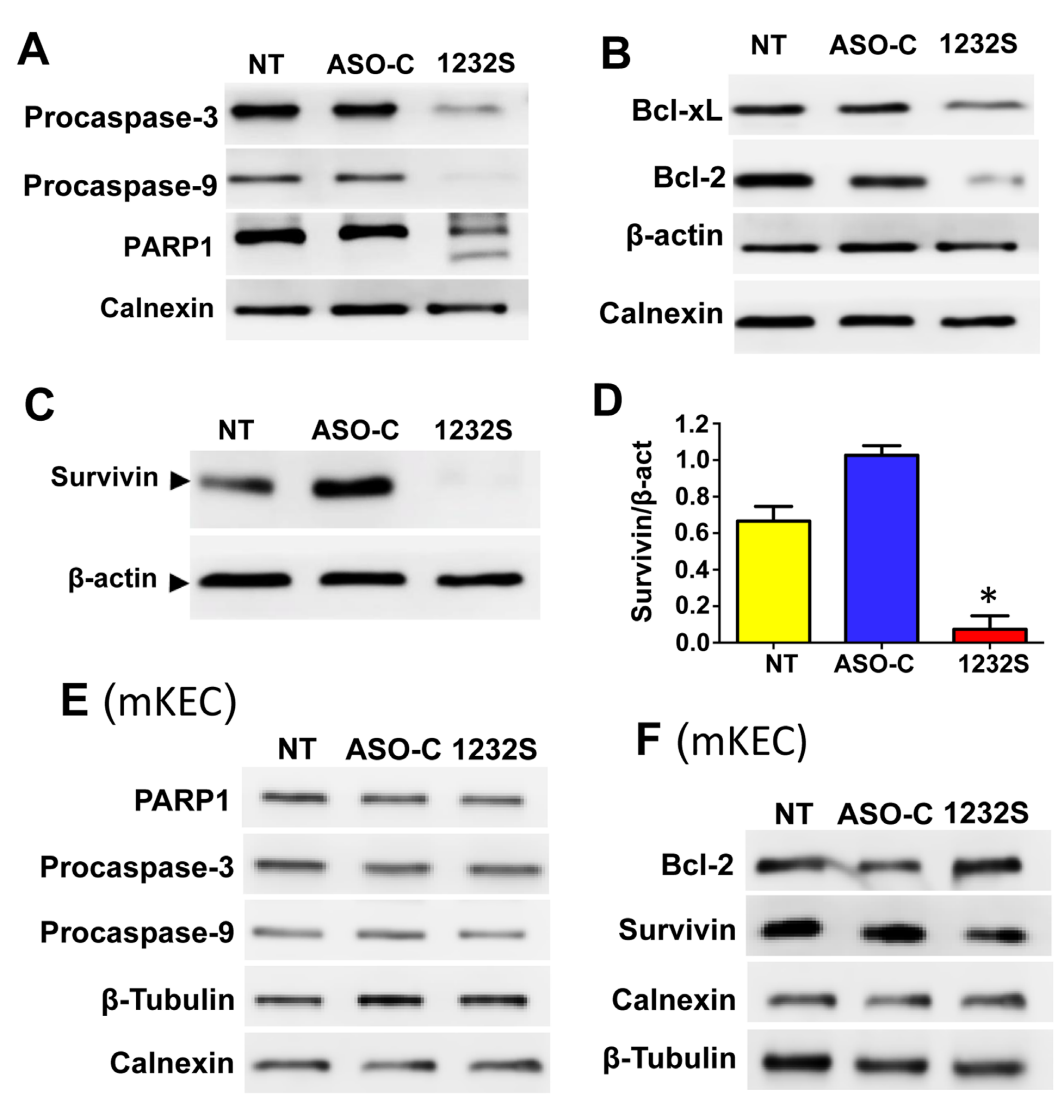
ASK induces activation of pro-apopototic factors and downregulation of anti-apoptotic factors **A**. RenCa cells were transfected with 100 nM ASO-C or ASO-1232S or left untreated (NT) for 48 h, harvested and processed for Western blot using calnexin as loading control. ASK induces processing of procaspase-3, procaspase-9 and PARP1. **B**. ASK induces downregulation of the anti-apoptotic factors Bcl-xL and Bcl-2 compared to controls; β-actin and calnexin were used as loading controls. **C**. RenCa cells treated as in (A) for 24 h displayed a drastic reduction in survivin, compared to controls, which was around 85% as evidenced by a triplicate densitometric analysis (**D**.; *p < 0.005). **E.** Western blot analysis of mKEC cells treated as in (A) for 48 h showed that ASK did not affect PARP1, procaspase-3 or procaspase-9, compared to controls. **F**. ASK did not induce downregulation of Bcl-2 or Survivin in mKEC cells. In E and F, calnexin and β-tubulin were used as loading controls.

ASK also induces activation of apoptotic factors and downregulation of anti-apoptotic factors. RenCa cells were treated as before (Figure 3A) for 48 h and Western blot analysis indicate that pro-caspase-3 and -9 and PARP1 were processed after ASK (Figure 4A). In addition, the anti-apoptotic factors Bcl-xL and Bcl-2 were also downregulated by ASK (Figure 4B). Similarly, another anti-apoptotic factor, survivin, was also strongly downregulated after ASK (Figure 4C, 4D).

Figure 2C shows that ASK does not induce cell death of mKEC cells according to permeability to PI. In addition, ASK does not induce processing of PARP1, procaspase-3 or procaspase-9 after 48 h of treatment (Figure 4E). Similarly, PARP1, Bcl-2 and Survivin are not reduced ASK in these cells (Figure 4F).

### ASK induces inhibition of colony formation and cell invasion

**Figure 5:**
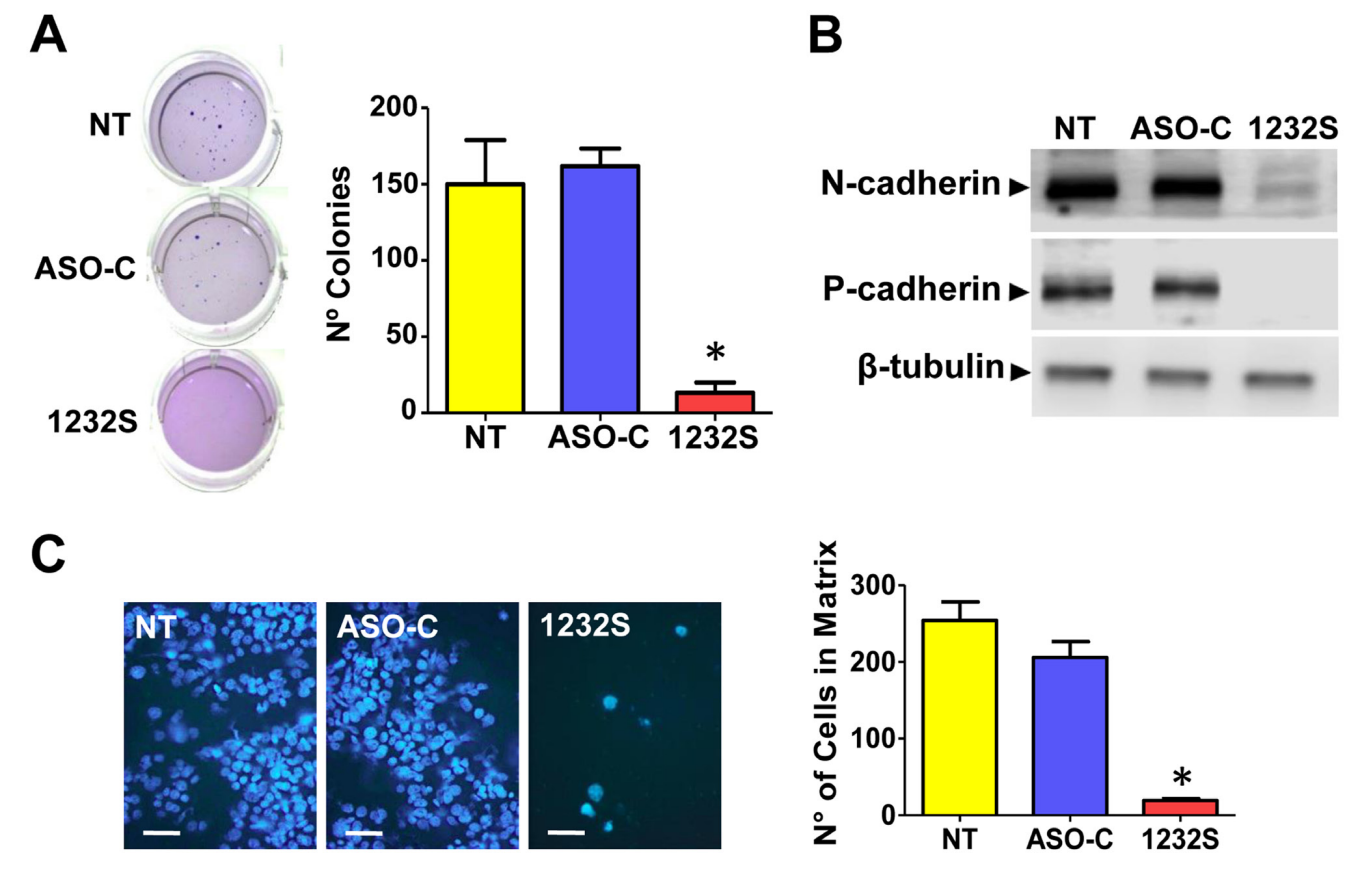
ASK inhibits tumorogenic and metastatic potential of RenCa cells **A**. RenCa cells were transfected with 100 nM ASO-C or ASO-1232S or left untreated (NT) for 48 h. After harvesting and counting, 2,000 Tb-negative cells were seeded in soft agar (see Materials and Methods) and colonies >50 μm in diameter were scored at 2-3 weeks. A triplicate analysis showed that ASK inhibited colony formation by about 90% (*p < 0.005). **B**. ASK downregulates proteins involved in metastasis. RenCa cells were transfected as in (A) for 24 h and protein extracts were analyzed by Western blot with antibodies against N-cadherin and P-cadherin, using β-tubulin as loading control. Both cadherins were strongly downregulated by ASK. **C**. ASK inhibits invasiveness of RenCa cells. Cells (100,000) treated as in (A) for 48 h were grown over Matrigel-coated inserts. After 24 h, inserts were fixed in cold methanol, stained with DAPI and mounted in Mowiol. At least 10 fields were evaluated by fluorescence microscopy (Bars = 25 μm). A triplicate analysis showed that ASK induced an inhibition of around 90% in invasiveness of RenCa cells (*p < 0,001).

A noteworthy property of transformed cells is the capacity of anchorage-independent growth and colony formation is considered a parameter of tumorigenesis [31]. RenCa cells were transfected with ASO-1232S or ASO-C or left untreated (NT) for 48 h. Cells were harvested, counted and 1000 Trypan blue-negative cells were seeded in soft agar in 12-well plates as described (see Materials and Methods). After 2-3 weeks in culture, colonies over 50 μm in diameter were counted and the results show that ASK induces a drastic inhibition of colony formation (Figure 5A).

To determine whether ASK affects the invasion capacity of RenCa cells, cells were transfected with ASO-C, ASO-1232S or left untreated for 24 h (see Materials and Methods). ASK induced a significant reduction in the expression levels of the epithelial-mesenchymal transition (EMT) markers, N-cadherin [32] and P-cadherin [33] (Figure 5B), suggesting that the treatment negatively affects metastatic capacity. This was reflected by a lower invasive potential as observed when cells were seeded onto Matrigel [14] after a 24 h treatment as above. ASK induced around 80% inhibition of invasion, compared to controls (Figure 5C).

### ASK *in vivo* induces reversal of RenCa tumor growth and increased survival

**Figure 6:**
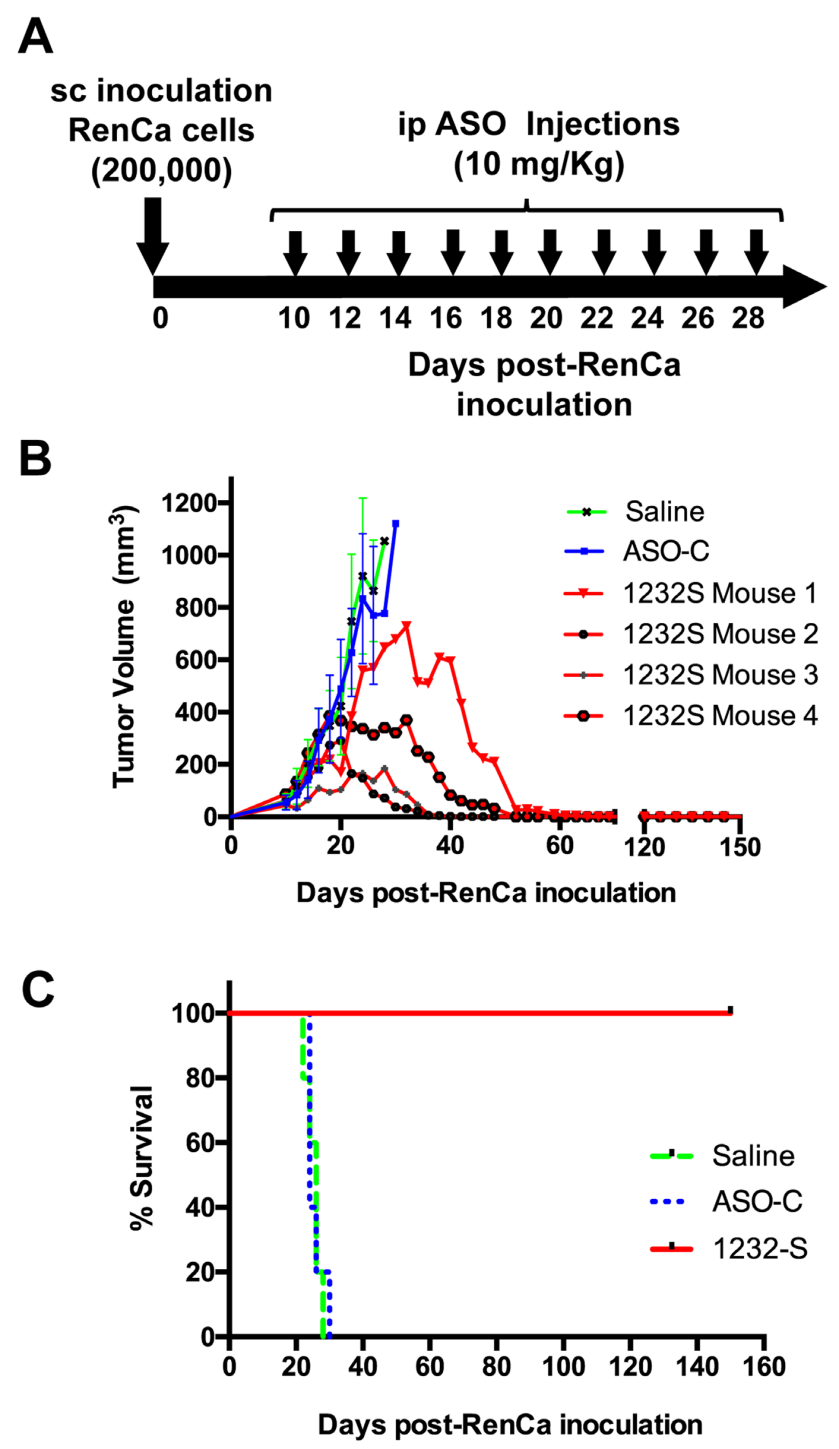
ASK inhibits subcutaneous RenCa tumor growth **A**. Experimental scheme. **B**. Twelve Balb/C mice were inoculated sc with 200,000 RenCa cells. At 10 days post-cell inoculation, mice harbored tumors of about 100 mm^3^. Animals were randomized into 3 groups of 4 mice, which received 10 ip injections of 200 μl saline alone or containing 200 μg ASO-C or ASO-1232S. At day 26 post-cell inoculation, the control groups reached tumors over 1000 mm^3^ and were sacrificed. The remaining mice reached different tumor size followed by progressive decrease of tumor volume. These four mice were healthy at day 150 post-cell inoculation. **C**. Kaplan-Meier survival curve. Saline and ASO-C control groups displayed 24 and 26-day mean survival, respectively, whereas all ASO-1232S-treated mice were alive and healthy at 150 days post-cell inoculation (*p* = 0.00051).

The above results suggest ASK as a strategy for *in vivo* treatment of renal cancer. Therefore, we performed ASK *in vivo* using a subcutaneous (sc) syngeneic model of RCC with immunocompetent Balb/C mice (Figure 6A). RenCa cells (200,000 cells/mouse) were inoculated subcutaneously on the left flank of Balb/C mice (see Materials and Methods). On day 10 post-cell inoculation, mice with tumors of about 100 mm^3^ were randomized into three groups of 4 mice each (Figure 6B), which were injected intraperitoneally (ip), in a blinded fashion, every other day with 10 doses of 250 μl saline alone or containing 200 μg ASO-C or ASO-1232S (Figure 6B). In one representative assay, on day 28 post-cell inoculation both control groups displayed tumors between 1,000 and 1,200 mm^3^ and were euthanized (Figure 6B). The ASO-1232S-treated group reached a maximum tumor growth of around 700 mm^3^ at day 32 and afterwards tumor size progressively diminished in size and disappeared in all mice from this group (Figure 6B). The Kaplan-Meier analysis shows 24 and 26-day median survival for saline and ASO-C groups, respectively, whereas all mice treated with ASO-1232S were alive and healthy at 150 days post-cell inoculation (Figure 6C), when the experiment was terminated.

**Figure 7:**
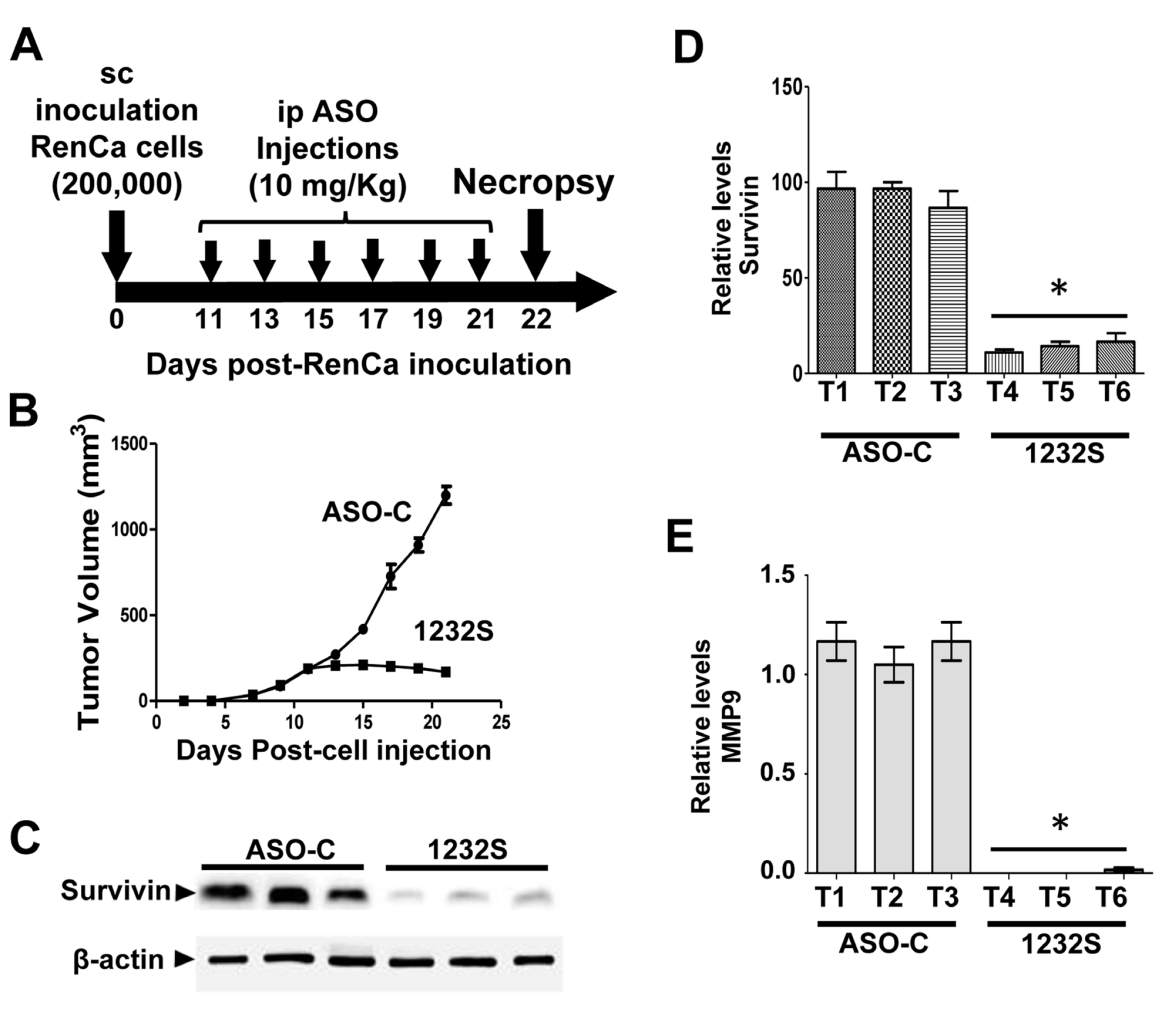
ASK *in vivo* induces downregulation of survivin and MMP9 Eight Balb/c mice were inoculated sc with 200,000 RenCa cells. Eleven days post-cell inoculation, mice contained tumors of about 200 mm^3^ and were randomized into 2 groups of 4 mice, which were treated by 6 ip injections on days 11, 13, 15, 17, 19 and 21 with 200 μg ASO-C or ASO-1232S in 200 μl saline. The day after the last injection, mice were euthanized and tumors were harvested and processed for Western blot. **A**. Scheme of the experiment. **B**. Growth curve of sc tumors, showing a drastic retardation in ASO-1232S treated mice, compared to controls. **C**. Representative blot of survivin in lysates of 3 tumors from each group. Tumors treated with ASO-1232S (T4, T5 and T6) exhibited downregulation of survivin, compared to ASO-C tumors (T1, T2 and T3). **D**. A triplicate analysis showing that ASK induced about 80% downregulation of survivin (*p < 0,001). **E**. Drastic downregulation of MMP-9 induced by ASK (*p < 0.0005).

To determine the effects of ASK *in vivo* at the molecular level, a second assay was carried out as described before by sc injection of 8 Balb/C mice with 200,000 RenCa cells. When tumor volumes reached about 200 mm^3^, mice were randomized into 2 groups which were injected ip on days 11, 13, 15, 17, 19 and 21 with 200 μg ASO-C or ASO-1232S in 250 μl saline (Figure 7A). The day after the last injection, mice were sacrificed and tumors were collected to prepare lysates. Mice treated with ASO-1232S displayed a strong retardation of tumor growth, compared to the control group (Figure 7B). Western blot of the tumors from ASO-1232S-treated mice showed downregulation of survivin compared to the control group (Figure 7C, 7D). Matrix metallopeptidase 9 (MMP-9), another factor, important in metastatic potential of tumor cells [34], was also strongly downregulated (Figure 7E).

### Orthotopic studies

**Figure 8:**
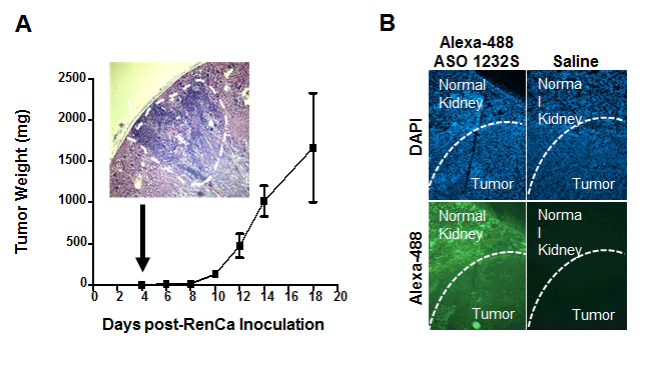
Kinetics of orthotopic RenCa tumor growth and tumoral 1232S entry **A**. Twenty one Balb/C mice were inoculated into the subcapsular structure of the kidney with 100,000 RenCa cells. Three mice each were sacrificed on days 4, 6, 8, 10, 12, 14 and 18 and kidneys were fixed. Four sections from each kidney were used to evaluate tumor growth. At day 4, 1-2 mm^3^ tumors were found (insert, magnification 4X). **B**. Incorporation of ASO-1232S into orthotopic tumors. RenCa cells (100,000) were injected into the subcapsular region of the left kidney of 2 Balb/C mice and sutured. On day 12 post-cell inoculation, one mouse was injected ip with 100 μg ASO-1232S conjugated to Alexa Fluor 488 and the other mouse was injected with saline. Two h after injection, mice were sacrificed, kidneys were collected, frozen at -70°C and sectioned with a cryostat. Sections were counterstained with DAPI and analyzed by fluorescence microscopy. Green fluorescence was found in the kidney parenchyma and in the tumor only of the mouse injected with Alexa 488-ASO-1232S (magnification 4X).

The results in Figure 6 and 7 provide a strong proof-of-concept supporting the anti-tumor and anti-metastatic effect of ASO-1232S on RenCa cells *in vivo*. However, the sc model for this cell line does not reflect the renal microenvironment from which RenCa cells were derived. Therefore, we used an orthotopic model previously described [35–37]. As a first step, we determined RenCa tumor growth kinetics. Twenty one Balb/C mice were injected with 100,000 RenCa cells orthotopically into the subcapsular space of the left kidney. On days 4, 6, 8, 10, 12, 14 and 18, three mice were euthanized and both kidneys were removed, weighed and fixed. Four sections were obtained from each left kidney and stained with H&E. At day 4 post-cell inoculation, tumors between 2 and 3 mm^3^ were detected in the left kidney, growing progressively until day 18 (Figure 8A). To determine whether ASO-1232S enters the tumor *in vivo*, mice with orthotopic RenCa tumors were injected once ip with 100 μg Alexa-488-labeled ASO-1232S at day 12 post-cell inoculation. After 2 h, mice were sacrificed and the left kidney was frozen and cryostat-sectioned. The normal kidney parenchyma and the solid RenCa tumor appeared fluorescent, confirming entry of Alexa-488-ASO-1232S into the tissue (Figure 8B).

**Figure 9:**
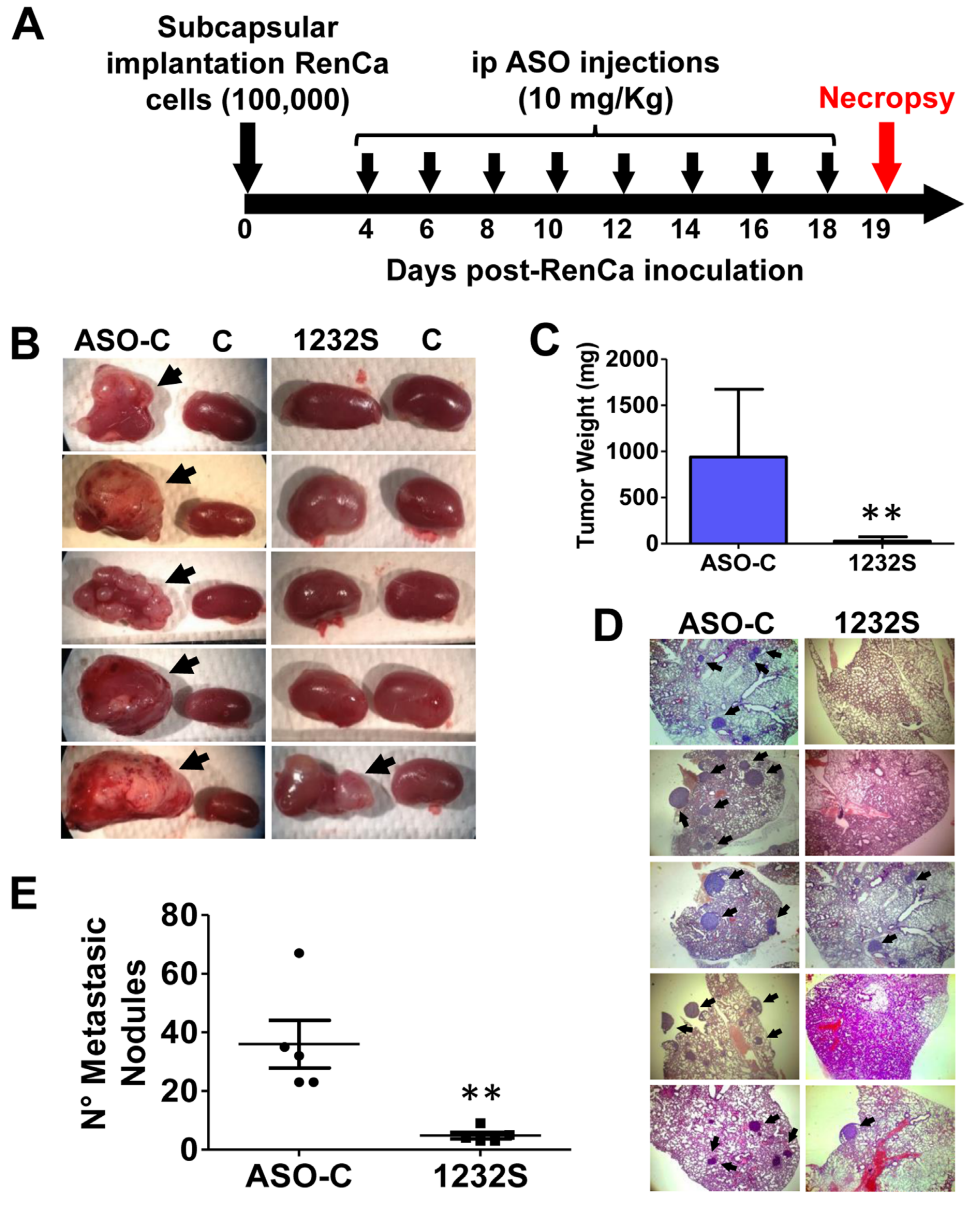
ASK *in vivo* inhibits primary tumor growth and lung metastasis in an orthotopic renal cancer model **A**. Scheme of the orthotopic protocol. Ten female Balb/C mice were injected under the renal capsule with 100 μl saline containing 100,000 RenCa cells and sutured. At day 4 post-cell inoculation, mice were randomized into 2 groups of 5 mice each, receiving 8 ip injections on days 4, 6, 8, 10, 12, 14, 16 and 18 of 250 μl saline containing 200 μg of ASO-C or ASO-1232S. On day 19 post-cell inoculation, mice were sacrificed under anesthesia and both kidneys and lungs were resected and fixed. Kidneys were weighed before fixing. **B**. Macroscopic presence of kidney tumors comparing the left and the right (control) kidney (arrows) of each mouse. Only one mouse from the ASO-1232S group developed a small tumor, whereas all 5 mice form the ASO-C group displayed large tumors. **C**. Tumor weight of the left kidney, comparing ASO-C and ASO-1232S (**p < 0.005). **D**. Four sections from each fixed lung were stained with H&E and metastatic nodules (arrows) were microscopically scored. The figure shows representative images from the left kidney of each mouse (magnification 4X). **E**. ASK induces a low number of metastatic nodules as compared to the control ASO-C (**p < 0.005).

In order to determine the *in vivo* effect of ASO-1232S on orthotopic tumors, 10 Balb/C mice were inoculated as described above. After 4 days, the animals were randomized into 2 groups of 5 mice each and ip injected in a blinded fashion on days 4, 6, 8, 10, 12, 14, 16 and 18 with 250 μl saline containing 200 μg of either ASO-C (control) or ASO-1232S (Figure 9A). On day 19 post-cell inoculation, mice were euthanized and the lungs and both kidneys were removed and fixed. In one representative assay and, in contrast to the control group which contained large renal tumors, 4 mice treated with ASO-1232S showed total absence of tumor, while one mouse contained a small tumor (Figure 9B, arrows). Tumor weight was estimated subtracting the right kidney from the left kidney showing that ASK was higly effective in inhibiting tumor growth (Figure 9C). The number of lung metastatic nodules in animals treated with ASO-1232S was considerably and significantly lower than the controls (Figure 9D, 9E).

### Metastasis assay

**Figure 10:**
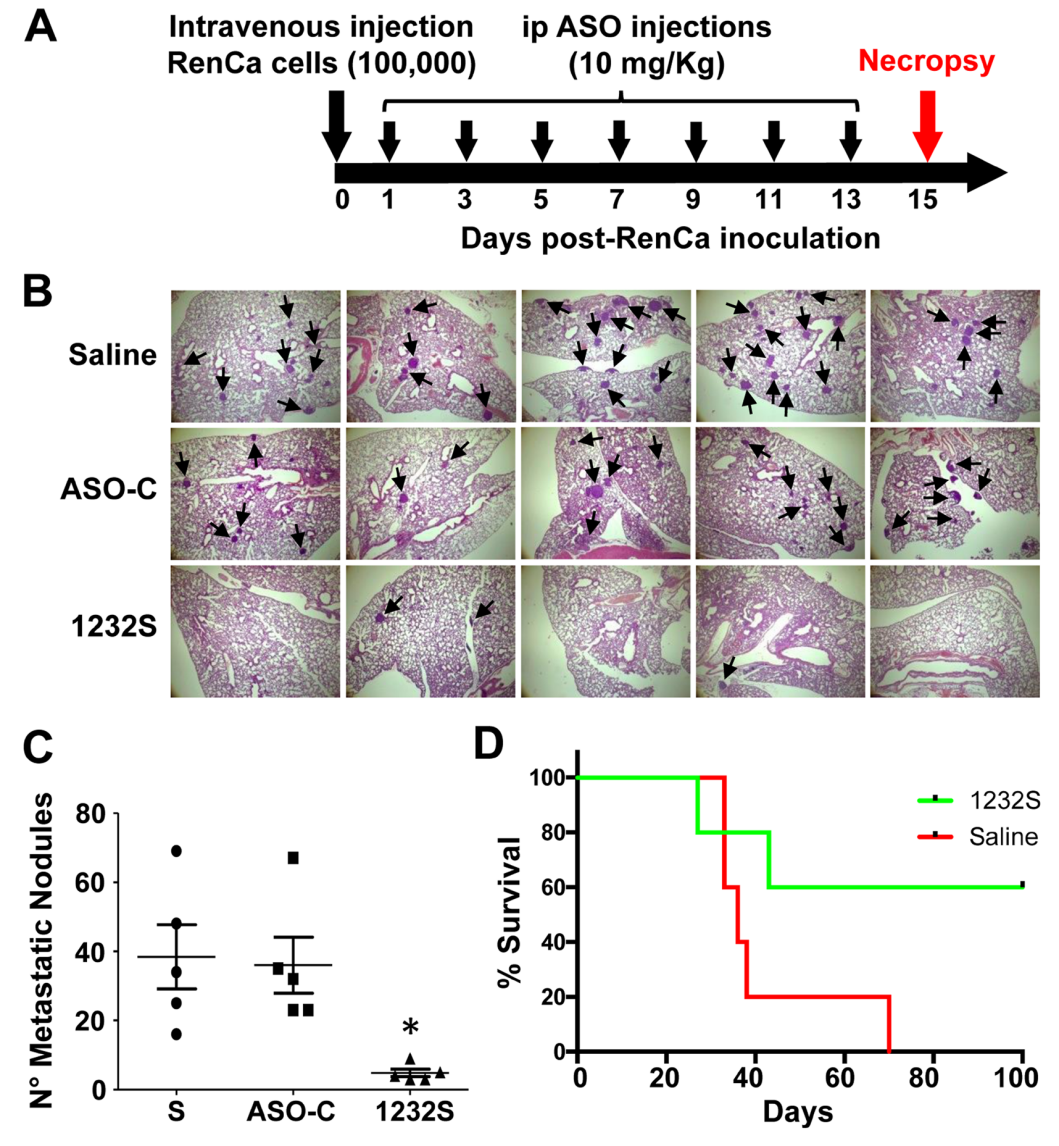
Tail vein RenCa metastasis assay **A**. Scheme of the metastasis protocol. Fifteen female Balb/c mice were inoculated through the tail vein with 100 μl saline containing 100,000 RenCa cells and animals were randomized into 3 groups of 5 mice each, which received 7 ip injections on days 1, 3, 5, 7, 9, 11 and 13 of 250 μl saline alone (S) or containing 200 μg ASO-C or ASO-1232S. On day 15, mice were sacrificed and lungs were excised, fixed and sections stained with H&E. **B**. Saline and ASO-C-treated mice harbored abundant metastatic nodules (arrows) in the lungs, whereas the ASK group displayed considerably fewer nodules (magnification 4X). **C**. Metastatic nodule count depicts an important and statistically significant reduction in the number of metastatic nodules in the ASK group, compared to both controls (*p < 0.005). **D**. Metastasis survival assay. Ten mice were inoculated with 100,000 RenCa cells as described in (A). On day 1 post-cell inoculation, mice were randomized into 2 groups of 5 mice each, which were injected ip with 200 μg ASO-C or ASO-1232S in 250 μl saline, on days 1, 3, 5, 7, 9, 11 and 13. Tumor growth and survival was monitored until day 100 post-cell inoculation (*p* = 0.07).

To directly assess the effect of ASO-1232S on metastasis, we performed a “classic” *in vivo* metastasis assay [38]. Fifteen Balb/C mice were injected through the tail vein with 100,000 RenCa cells in 100 μl sterile saline. Mice were then divided into 3 groups, which received 7 ip injections on days 1, 3, 5, 7, 9, 11 and 13 of 250 μl sterile saline alone, or containing 200 μg ASO-C or ASO-1232S (Figure 10A). On day 15, mice were euthanized and lungs were collected and fixed (Figure 10A). In one representative result, lungs of ASO-1232S-treated mice displayed a considerably and significantly lower number and size of lung metastatic nodules, compared to both control groups (Figure 10B, 10C). In a parallel tail-vein metastasis assay, ASO-1232S treatment induced 60% survival for 100 days, compared to the saline control group (Figure 10D).

## DISCUSSION

These results corroborate our previous proposal that downregulation of the ASncmtRNAs seems to be a general hallmark of cancer [12, 14–16]. The mechanism underlying downregulation of the ASncmtRNAs is not clear, but we determined the fate of these transcripts in human foreskin keratinocytes immortalized with Human Papilloma virus (HPV). The results indicated that the E2 viral oncogene of high risk HPV 16 and HPV 18 induces downregulation of the ASncmtRNAs [13].

Here we show that knockdown of the ASncmtRNAs or ASK induces RenCa cell death by classical hallmarks of apoptosis *in vitro* and *in vivo*. Hypothetically, the lower copy number of ASncmtRNA in tumor cells renders them more vulnerable to ASK. Indeed, the same treatment does not affect viability of normal murine epithelial cells (mKEC), as observed previously with other mouse and human normal cells [14, 16], supporting the selective and innocuous nature of this approach.

An intriguing issue is why the ASncmtRNAs are not suppressed completely in cancer cells. Previously we proposed that these transcripts may function as tumor suppressors [11–13] and perhaps the low copy number of ASncmtRNAs may be crucial to override cell cycle control mechanisms and thus to fuel cancer progression, by switching to a pro-survival function. This switch from tumor suppressor to oncogene function has been described before. For example, the missense mutation of p53 (mp53), existing in approximately 50% of human cancers, confers oncogenic properties to mp53 that contribute to cancer progression and metastasis [39–41].

Apoptosis of RenCa cells induced by ASK is potentiated by downregulation of survivin *in vitro* (Figure 4C, 4D) and *in vivo* (Figure 7C, 7D), and Bcl-2 and Bcl-xL, members of another family of anti-apoptotic factors (Figure 4B) [42]. Similar results were obtained with human tumor cell lines and the B16F10 murine melanoma cell line [14, 16]. ASK also induces molecular effects which result detrimental to metastatic potential, such as downregulation of N-cadherin, P-cadherin (Figure 5B) and MMP9 (Figure 7E), further strengthening the potential of this strategy for RCC therapy. To explain this observed pleiotropic effect in RenCa cells, we have proposed that ASK induces upregulation of microRNAs (miRs) derived from the ASncmtRNAs [16, 43, 44].

Our *in vivo* studies in a sc syngeneic model of RenCa cells show complete reversal of tumor growth (Figure 6B). At 150 days post-cell inoculation, animals appeared healthy, in contrast to mice treated with saline or ASO-C. Next, we performed an orthotopic assay of murine RCC by injection of RenCa cells into the subcapsular region of the left kidney. Treatment commenced at 4 days post-cell inoculationy and on day 19, mice were euthanized and both kidneys were recovered together with lungs and fixed. Macroscopic analysis showed that all the control mice contained tumors of different size (Figure 9B, 9C). In contrast, only one mouse treated with ASO-1232S exhibited a small tumor (Figure 9B). Histological analysis of four sections from each lung showed that control mice treated with ASO-C contained several and large metastatic nodules (Figure 9D, 9E). In contrast, only two lungs of mice treated with ASO-1232S contained metastatic nodules, which were significantly fewer and smaller (Figure 9D, 9E).

Tail vein injection of RenCa cells also demonstrated the potent effect of ASK to inhibit lung metastasis. ASK significantly reduced the number of metastatic nodules in the lungs (Figure 10B, 10C). In addition, a similar assay showed that ASK induces 60% survival of mice (3 out of 5) until 100 days post-cell inoculation (see Figure 10D), when the experiment was finalized. Metastasis is a complex process that requires several factors (proteins and ncRNAs) that cooperatively induce the invasive capacity of tumor cells. Among these factors, survivin, besides its participation as an anti-apoptotic factor and in cell division, is an active player in metastasis [21]. On the other hand, epithelial-mesenchymal transition (EMT) requires a switch from E-cadherin to N-cadherin [45–49]. Here we show that ASK induces inhibition of the invasive capacity of RenCa cells (Figure 5C), together with downregulation of survivin (Figure 4C, 4D), N-cadherin, P-cadherin (Figure 5B) and MMP9 (Figure 7E), explaining the decrease in metastatic potential of RenCa cells *in vivo*.

One of the main difficulties in treating mRCC, besides its chemo- and radioresistance, is its established intratumoral and interpersonal heterogeneity [50]. One of the advantages of ASK is that the observed effects on tumor cells are independent of the genetic background of the tumor. Thus, ASK induces over 60% cell death *in vitro* in human renal cancer cell lines with wild type (Caki-1 cells) and mutant von Hippel-Lindau tumor suppressor gene (A498 cells), as well as in patient-derived primary cultures of clear cell RCC (V. Borgna, unpublished data).

The present pre-clinical results with RenCa cells, together with our previous results using the B16F10 murine melanoma model, establish proof-of-concept that the ASncmtRNAs may constitute a potent and selective target to develop a treatment for different types of cancer and positions this approach as an attractive strategy ready for clinical testing. In this respect, the USA Food and Drug Administration (FDA) approved an oligonucleotide directed to the human ASncmtRNAs as IND for a Phase I Clinical Trial for solid tumors, which is currently under way and close to completion at UCSF, California, USA.

## MATERIALS AND METHODS

### Animal studies

Animal studies were conducted in accordance with the guidelines of Comisión Nacional de Investigación Científica y Tecnológica (Conicyt), Chile and approved by the Ethical Committee of Fundación Ciencia & Vida. Balb/C mice 6-8 weeks of age were obtained from The Jackson Laboratory, (Bar Harbor, ME, USA) and maintained under specific pathogen-free conditions and maintained in a temperature-controlled room with 12/12 h light/dark schedule with food and water *ad libitum*.

### Cell culture

RenCa cells, a murine renal adenocarcinoma cell line, have been used extensively as a syngeneic model to test new therapeutic drugs for RCC. In addition, these *in vivo* preclinical studies in normal and immunocompetent mice would have a better impact to be extrapolated to human RCC. RenCa cells were purchased from ATCC (CRL-2947) (Manassas, VI, USA) and cultured according to ATCC guidelines. Upon arrival, cells were expanded and frozen in liquid nitrogen at low passage number. After resucitation, cells were not passaged beyond 6 months. Cultures were checked periodically for mycoplasma using the EZ-PCR Mycoplasma Test Kit (Biological Industries, Beit Haemex Ltd, Israel). To obtain primary cultures of mouse kidney epithelial cells, the renal capsule was removed and 1-2 mm^3^ pieces of the renal cortex were dissected. The tissue was washed three times in PBS at room temperature and digested for 4 h at 37°C in RPMI containing 1 mg/ml collagenase I, 2 mg/ml collagenase IV, 1 mg/ml Dispase, 20 μg/ml hyaluronidase and 2000 U/ml DNase I. The cell suspension was centrifuged at 200 x g for 5 min at RT and the pellet was suspended in PBS and again centrifuged for 5 min at 200 x g. The final pellet was suspended in RPMI containing 5% FBS and seeded on collagen I-coated T25 flasks (Nunc, Walthan, MA, USA). Cells were incubated at 37°C and 5% CO_2_. Fibroblasts were removed by differential trypsinization [51, 52].

### *In situ* hybridization

To detect the mSncmtRNA and the ASncmtRNAs, FISH was performed as described [11, 12] on murine kidney epithelial cells and RenCa cells using antisense probe (5’AATAGGATTGCGCTGTTATCCCTA) and sense probe (5’TAGGGATAACAGCGCAATCCTATT), respectively, coupled to Texas red at its 5’ end (Molecular Probes, Eugene, OR, USA).

### Cell transfection

ASOs used in this study were synthesized by BioSearch Inc. (Novato, California, USA) with 100% phosphorothioate (PS) internucleosidic linkages. The ASOs utilized were ASO-1232S (5’ CCTAACGAGCTTGGTGATAGC) and control ASO (ASO-C) (5’AGGTGGAGTGGATTGGGG). Transfection was carried out with 2 μg/ml Lipofectamine2000 (Invitrogen, Carlsbad, CA, USA), according to manufacturer´s directions and using 100 nM of each ASO dissolved in sterile saline.

### Proliferation and cell viability

Cell count was performed by Trypan blue (Tb) or propidium iodide (PI) exclusion. PI was added at 1 µg/ml 1 min before flow cytometry on a BD FACS Canto Flow Cytometer (San Jose, CA, USA). For Tb, the number of viable cells was determined counting at least 100 cells per sample in triplicate in a hemocytometer [14–16].

### Conventional RT-PCR amplification

RNA was extracted with TRIzol Reagent (Invitrogen, Carlsbad, CA, USA) as described [11–14, 16]. To eliminate mtDNA contamination, RNA was treated with TURBO DNA-free (Ambion, Waltham, MA, USA) according to manufacturer's instructions. Reverse transcription was carried out with 100 ng RNA, 100 ng random hexamers for 18S rRNA or 20 pmoles of gene-specific primer (5’ CCTTACAAATAAGCGCTCTCAAC) for ASncmtRNAs, 0.5 mM each dNTP, 10 mM DTT, and 100U MMLV (Invitrogen, Carlsbad, CA, USA). Reactions were incubated at 25°C for 10 min, 37°C for 60 min and 75°C for 5 min. PCR was carried out in 50 µl containing 2.5 µl cDNA, 0.4 mM each dNTP, 0.8 µM each primer, 3 mM MgCl_2_ and 2 U GoTaq (Promega, Madison, WI, USA). The sequences of the primers used were 5’ ACCGTGCAAAGGTAGCATAATC (for) and 5’ ATATATACGTACAACCTTCTCTAGG (rev) for mASncmtRNA-1, 5’ ACCGTGCAAAGGTAGCATAATC (for) and 5’ TTAAACCTAATAACCTTCTCTAGG (rev) for mASncmtRNA-2 and 5’ GTAACCCGTTGAACCCCATT (for) and 5’ CATCCAATCGGTAGTAGCG (rev) for 18S rRNA.

### Mitochondrial membrane depolarization

After seeding 50,000 cells/well in 12-well plates, cells were transfected the next day for 24 h as described above. Afterwards, cells were loaded with 20 nM tetramethylrhodamine methyl ester (TMRM, Molecular Probes, Eugene, OR, USA) for 15 minutes at 37°C [14, 16], harvested and analyzed by flow cytometry on a BD-FACS Canto Flow Cytometer. As positive control, mitochondrial depolarization was elicited using 10 µM of staurosporine (STP).

### Western blot

Cells transfected for 24 h were harvested, washed in ice-cold PBS and sedimented at 1,000 x g for 10 min at RT. Pellets were suspended in RIPA buffer (10 mM Tris-HCl pH 7.4, 1% sodium deoxycholate, 1% Triton X-100, 0.1% sodium dodecyl sulfate), containing 1 mM PMSF and protease inhibitor cocktail (Sigma-Aldrich, St. Louis, MS, USA). Protein concentration was quantified with the Bradford microplate system Gen5TM EPOCH (BioTeK, Winooski, VE, USA) (13). Proteins (30 μg/lane) were resolved by SDS-PAGE and transferred to polyvinylidene difluoride (PVDF) membranes. Membranes were probed with antibodies against survivin (rabbit polyclonal; 1:1,000), caspase-3 (rabbit polyclonal; 1:1,000) or caspase-9 (mouse monoclonal; 1:1,000), from Abcam (Cambridge, MA, USA); PARP1 (rabbit polyclonal 1:300), β-tubulin (rabbit polyclonal; 1: 1,000), Bcl-xL, Bcl-2 (mouse monoclonal, 1:1,000) from Cell Signaling (Danvers, MA, USA); N-cadherin 1:1,000 (rabbit polyclonal, Thermo Fisher Scientific, Waltham, MA, USA); P-cadherin (rabbit polyclonal 1:500, Santa Cruz, Biotechnology, Santa Cruz, CA, USA); calnexin (mouse monoclonal, 1:1,000, Novus, Littleton, CO, USA) or β-actin (mouse monoclonal, 1:4,000, Sigma-Aldrich, St. Louis, MS, USA). Primary antibodies were detected using peroxidase-labeled anti-mouse or anti-rabbit IgG (1:5,000, Calbiochem, Billerica, MA, USA). Blots were detected with the EZ-ECL system (Biological Industries, Beit Haemex, Israel). The intensity of each band was quantified using ImageJ software (NIH).

### TUNEL assay

The TUNEL procedure was performed using the Dead-End Fluorometric TUNEL kit (Promega, Madison, WI, USA) [14, 16], according to manufacturer's instructions. As positive control, cells were incubated with STP as described above. Samples were analyzed on an Olympus BX-53 fluorescence microscope.

### Determination of phosphatidylserine exposure

Phosphatidylserine (PS) exposure was determined by Annexin-V binding with the APOtarget kit (Invitrogen, Carlsbad, CA, USA), according to manufacturer's directions and analyzed by Flow cytometry or fluorescence microscopy [14, 16].

### Colony formation and invasion assay

Anchorage-independent cell growth was determined by colony formation in soft agar as described [14, 16]. Untreated RenCa cells (NT) or cells transfected with 100 nM ASO-C or ASO-1232S for 48 h were harvested and 2,000 Tb-negative cells were seeded into 12 well-plates in soft agar. Formation of colonies >50 μm in diameter were scored at 2-3 weeks. For matrigel invasion assay, 10^5^ cells treated as above for 48 h were seeded over Matrigel-coated inserts (Matrigel Invasion Chamber 8.0 μm; BD Biosciences, San Jose, CA, USA). After 24 h, inserts were fixed in cold methanol, stained with DAPI and membranes were mounted in Mowiol, and 10 fields per sample were evaluated under an Olympus BX-53 fluorescence microscope at 40x.

### Subcutaneous model

To determine the antitumor effect of ASO-1232S, 12 Balb/C mice were routinely injected subcutaneously (sc) with 2 × 10^5^ RenCa cells on the right flank. When tumors reached a volume of about 100 mm^3^, mice were randomized into three groups of 4 animals, which received 10 intraperitoneal (ip) injections with 250 μl saline, or 200 μg ASO-C or ASO-1232S in 250 μl of saline every other day. Tumor growth was monitored every other day with a caliper and tumor volumes were calculated on the basis of the formula: tumor volume = length x width x height x 0.5236.

### Orthotopic assay

To determine tumor growth kinetics in the kidney, 21 Balb/c mice were injected with 100,000 RenCa cells orthotopically into the subcapsular space of the left kidney [35–37]. On days 4, 6, 8, 10 and 12, 3 mice were sacrificed under anesthesia and both kidneys were removed and fixed. Four sections were obtained from the left kidney and stained with H&E. Four days post-cell inoculation, tumors between 2-3 mm^3^ were detected in the kidney, after which they grew progressively in size (Figure 8A). Notice that ASO-1232S conjugated to Alexa-448 enters the kidney and the RenCa tumor *in vivo* (Figure 8B). Next, 10 Balb/C mice were injected orthotopically with 100,000 RenCa cells in the subcapsular space of the left kidney. On day 4, animals were randomized into 2 groups of 5 mice each and ip injected in a blinded fashion on days 4, 6, 8, 10, 12, 14, 16 and 18 with 250 μl saline containing 200 μg ASO-C or ASO-1232S (Figure 9A). On day 19 post-cell inoculation, mice were euthanized under anesthesia and the lungs and the right and left kidneys were removed and fixed. Tumor weight was estimated from the difference between the left and right kidney. Four sections from each kidney were stained with H&E and analyzed under an Olympus BX-53 microscope.

### Metastasis assay

To directly assess the effect of ASO-1232S on metastasis, we performed an *in vivo* metastasis assay [38]. RenCa cells (100,000/animal) were injected through the tail vein of 15 Balb/C mice, in a total volume of 100 μl sterile saline. Mice were then divided in 3 groups which received 6 ip injections on days 1, 3, 5, 7, 9, 11 and 13, of 250 μl sterile saline alone or containing 200 μg ASO-C or ASO-1232S. On day 15, mice were euthanized and lungs were collected and fixed. Four sections from each lung were stained with H&E and evaluated for metastasic nodules.

### Statistical analysis

Statistical analysis was performed using Graph Pad Prism version 6.2. One-tailed t-tests and two-way ANOVA tests were used to determine significance between experimental groups. Kaplan-Meier method analysis with log rank sum test was utilized for *in vivo* survival studies. Significance (*p*-value) was set at the nominal level of *p* < 0.1.
